# Can Natural and Synthetic Zeolites Be Dietary Modulators of Microorganism Population, Fermentation Parameters, and Methane Emission in the Rumen? A Preliminary Study on Cow

**DOI:** 10.3390/molecules30204040

**Published:** 2025-10-10

**Authors:** Małgorzata P. Majewska, Renata Miltko, Anna Tuśnio, Marcin Barszcz, Kamil Gawin, Joanna Bochenek, Urszula Wolska-Świętlicka, Barbara Kowalik

**Affiliations:** The Kielanowski Institute of Animal Physiology and Nutrition, Polish Academy of Sciences, Instytucka 3, 05-110 Jabłonna, Poland; r.miltko@ifzz.pl (R.M.); a.tusnio@ifzz.pl (A.T.); m.barszcz@ifzz.pl (M.B.); k.gawin@ifzz.pl (K.G.); j.bochenek@ifzz.pl (J.B.); u.wolska-swietlicka@ifzz.pl (U.W.-Ś.); b.kowalik@ifzz.pl (B.K.)

**Keywords:** clinoptilolite, zeolite 4A, protozoa, bacteria, methanogens, short-chain fatty acids, ammonia, amines, heifers

## Abstract

Zeolites are ‘magic stones’ with crystalline structures and unique properties, which enable them to selectively adsorb molecules, including gases. The aim of the study was to determine the effect of different types and doses of zeolites on microorganisms, nutrient digestion, and methane production in the rumen. The study was conducted on five two-year-old Jersey heifers (350 kg live weight) fistulated to the rumen in a 5 × 5 Latin square design divided into five feeding groups: control (basal diet), ZN2 (+120 g clinoptilolite/d), ZS2 (+120 g ZP-4A zeolite/d), ZN4 (+240 g clinoptilolite/d), and ZS4 (+240 g ZP-4A zeolite/d). During five periods of the experiment, the samples of the ruminal fluid and digesta were taken before and 3 h after feeding. The pH value, bacteria and methanogens populations, as well as short-chain fatty acids (SCFAs) and methane production in the rumen were not affected after zeolite addition (*p* > 0.05). ZN2 diet decreased the number of total protozoa by 41.2% (*p* = 0.023) and *Entodinium* spp. by 51.1% (*p* = 0.021), while ZS2, ZN4, and ZS4 diets reduced *Diplodinium* population by 70.5% (*p* < 0.001) 3 h after feeding in comparison to the control diet. An increased population of *Ophryoscolex* spp. was noted in ZN2 and ZS4 cow 3 h after feeding (*p* < 0.001; 0.15 × 10^4^/mL and 0.08 × 10^4^/mL vs. 0.02 × 10^4^/mL) when compared to control animals. Furthermore, ZS4 diet increased ammonia (*p* = 0.007; 3.97 mM/L vs. 2.27 mM/L), tryptamine (*p* = 0.014; 0.009 µmol/g vs. 0.007 µmol/g) and 1.7-diaminoheptane (*p* < 0.001; 0.016 µmol/g vs. 0.006 µmol/g) concentrations in the rumen, while phenylethylamine level was 90.9% higher in ZN4 cows (*p* = 0.007), in comparison to control, depending on time. To summarise, zeolites may act in a type- and dose-dependent manner on the protozoa population and indicators of protein degradation.

## 1. Introduction

Recently, there has been an increasing interest in society in the issues related to global climate change, especially greenhouse gases. According to the report of the Intergovernmental Panel on Climate Change (IPCC), methane, as one of the greenhouse gases, accounts for 30% of global warming [1]. Livestock production is responsible for 35% of total anthropogenic methane emissions into the atmosphere, and ruminants are considered to be the main producers of this gas [1,2]. Interestingly, this phenomenon has harmful consequences not only for the natural environment but also for high-yielding cows, for which the excessive methane production is connected with a loss of about 2–12% of gross energy, depending on the feeding system [3]. Thus, scientists are seeking nutritional additives (especially of natural origin) that can be used in ruminant nutrition with the potential to limit methane production during enteric fermentation [4].

Zeolites, commonly known as ‘magic stones’, are hydrated aluminosilicates with a crystalline structure, in which a channel system and chambers can be distinguished [5]. These compounds constitute a diverse group with physicochemical properties, in which about 40 types of natural zeolites can be distinguished, of which clinoptilolite is the best known, and about 150 synthetic zeolites artificially produced in the autoclaves at high temperature and pressure [5]. In view of the unique properties, zeolites have been used in the industrial sector, ecology, and biomedical applications [6,7]. Their porous structure and large surface area of several hundred m^2^/g allow for high sorption capacity (‘molecular sieves’), ion-exchange ability as well as selectivity dependent on the shape and size of the molecules relative to the pore diameter [8,9]. Zeolites can adsorb and absorb gases, water, radioactive elements, toxic substances, heavy metals, and smells [10]. Most importantly, such porous materials are stable in the gastrointestinal tract and do not react with nutrients and body fluids [10,11]. For that reason, they are considered to be safe in both human and animal nutrition and constitute an attractive alternative to the plant extracts for growers because of their low cost.

Most microbiological studies on zeolites were carried out in laboratory conditions and were focused mainly on wastewater management and water purification [12,13]. Studies conducted on animals showed that zeolites are buffering agents and may improve fibre digestion [14,15,16], animal productivity [17,18], as well as animal health and immune status in newborn animals [19,20]. Interestingly, the results of recent laboratory studies may indicate a potential involvement of zeolites in hydrogen [21,22] and methane adsorption [23,24].

In light of these premises, the hypothesis of the study assumed that the zeolites may reduce methane production by modifying the number of microorganisms and carbohydrate fermentation in the rumen. Thus, the aim of the study was to determine and compare the effects of different types of zeolites (natural vs. synthetic) and their contribution in a diet on the number of bacteria, methanogens, and protozoa, as well as to measure the concentrations of short-chain fatty acids (SCFAs), ammonia, amines, and gas production in the rumen of the cow.

## 2. Results

### 2.1. Diets

All animals received balanced diets, which contained approximately 10% of crude protein, 30% of crude fibre, as well as 4% of crude fat (Table 1). The composition of the cow diet and feed intake were previously presented in the study of Majewska et al. [16] as a part of this experiment. It appeared that orts constituted approximately 1% of the total diet, depending on the feeding group (control, ZN2, ZS2, ZN4); however, in cows fed a higher dose of synthetic zeolites (ZS4 group), the feed residues were more than 5 times greater (up to 5.23%).

### 2.2. Microorganisms

Significant interaction between diet and sampling time for all types of ciliates was noted (*p* ≤ 0.05, Table 2). ZS2 and ZN2 diets decreased total protozoa number by 36.7% (*p* = 0.048) and 41.2% (*p* = 0.021) in comparison to the control group, respectively, depending on the sampling time. An upward trend of total protozoa number was noted in ZS4 animals in comparison to the ZN2 group (*p* = 0.058, 17.60 × 10^4^/mL vs. 11.01 × 10^4^/mL) 3 h after feeding. Furthermore, a decreased number of *Entodinium* was noted in the ruminal fluid of ZN2 cow 3 h after feeding, when compared to control animals (*p* = 0.021; 6.88 × 10^4^/mL vs. 14.07 × 10^4^/mL). In addition, the ZS2 diet significantly reduced the number of *Entodinium* in comparison to the ZS4 diet before feeding (*p* = 0.021; 6.98 × 10^4^/mL vs. 14.83 × 10^4^/mL, respectively). Both types and doses of zeolites administered to cow diets significantly decreased *Diplodinium* number, but the largest decrease was noted in ZS4 group in comparison to the control group before feeding (*p* < 0.001; 0.38 × 10^4^/mL vs. 1.98 *×* 10^4^/mL) and 3 h after feeding (*p* < 0.001; 0.56 × 10^4^/mL vs. 2.07 × 10^4^/mL). Interestingly, the number of *Ophryoscolex* in the rumen was two times higher in the ZS2 diet than in other feeding groups (*p* < 0.001) before feeding, whereas it was 46.7% and 86.7% higher in the ZN2 diet 3 h after feeding when compared to the ZS4 and control groups (*p* < 0.001), respectively. In contrast, the 86.7% and 58.8% reduction in *Ophryoscolex* number after feeding was noted for control and ZS2 animals (*p* < 0.001), respectively. A tendency of an increased number of *Isotricha* was noted for the ZS2 group in comparison to the ZS4 diet before feeding (*p* = 0.058; 0.74 × 10^4^/mL vs. 0.46 × 10^4^/mL). Regarding the time effect, the number of *Isotricha* was 67.4% higher in the ZS4 group (*p* = 0.012), while it was 8.10% lower in the ZS2 group (*p* = 0.55) 3 h after feeding. A reduced number of *Dasytricha* was documented in the ZS4 group before feeding in comparison to the ZN4 diet (*p* = 0.027; 1.54 × 10^4^/mL vs. 2.90 × 10^4^/mL). Moreover, the *Dasytricha* population increased in the ZS4 cow by 92.2% 3 h after feeding in comparison to the time before feeding (*p* < 0.05). The reverse findings over time were noted in the ZN4 group but only as a tendency (*p* = 0.066; 2.90 × 10^4^/mL vs. 2.01 × 10^4^/mL).

Neither type of zeolites nor their contribution in a diet did not significantly affected the number of total bacteria and methanogens in the rumen (Table 3). Only a tendency of a lowered number of the total methanogens in ZN2 animal 3 h after feeding was noted (*p* = 0.059; 8.09 log_10_ rrs copies/g vs. 7.97 log_10_ rrs copies/g).

### 2.3. Ruminal Parameters

The pH value of the ruminal fluid of the cow ranged from 6.83 to 7.27 (Table 4). The type of zeolites and their contribution in the cow diet had no significant effect on its value both before and 3 h after feeding (*p* = 0.176). Regardless of the feeding group, a significant decrease in pH value by an average of 3.95% 3 h after feeding was noted (*p* < 0.001).

Experimental factors did not significantly affect the concentration of total SCFAs, acetic acid, propionic acid, and methane in the rumen (*p* > 0.05; Table 4). Only the sampling time affected specific SCFAs in the rumen. Briefly, an increased concentrations of butyric acid in control (*p* = 0.045; 0.75 mM/100 mL vs. 0.63 mM/100 mL) and ZS4 groups (*p* = 0.086; 0.81 mM/100 mL vs. 0.70 mM/100 mL) as well as valeric acid in control animals (*p* = 0.077; 0.11 mM/100 mL vs. 0.09 mM/100 mL) 3 h after feeding were observed. On the contrary, the concentration of isoacids was on average 17.1% lower in ZN2, ZS2, ZN4, and ZS4 cows (*p* < 0.001), after feeding.

Both factors (diet and time) had a significant effect on ammonia concentration in the ruminal fluid (*p* < 0.05; Table 4). The ZS4 diet increased ammonia concentration by 88.5% in comparison to the ZN2 diet (*p* = 0.010) before feeding, and 3 h after feeding by an average of 69.0% when compared to other feeding groups (*p* = 0.005). A tendency of lower ammonia concentration was noted for control (*p* = 0.072; 3.52 mM/L vs. 2.27 mM/L) and ZS2 (*p* = 0.086; 3.16 mM/L vs. 2.53 mM/L) groups 3 h after feeding.

### 2.4. Biogenic Amines

The effect of diet, time, and their interaction on phenylethylamine (*p* = 0.009) and 1.7-diaminoheptane concentrations (*p* < 0.001) has been shown (Table 5). Briefly, the ZN4 diet significantly increased phenylethylamine concentration in the rumen before feeding in comparison to the control (*p* = 0.006; 0.021 µmol/g vs. 0.011 µmol/g), ZS2 (*p* = 0.001; 0.021 µmol/g vs. 0.010 µmol/g), and ZS4 groups (*p* < 0.001; 0.021 µmol/g vs. 0.009 µmol/g). Additionally, the level of 1.7-diaminoheptane in ZS4 animals was 1.67 times higher before feeding in comparison to control, ZN2, ZS2, and ZN4 groups (*p* < 0.001). Likely, the ZS4 diet significantly increased the concentration of tryptamine by 28.6% in comparison to the control (*p* = 0.037) and ZN2 (*p* = 0.030) diets 3 h after feeding. Moreover, a tendency of an increased concentration of cadaverine in the ZS4 diet 3 h after feeding in comparison to the control group (*p* = 0.061; 0.060 µmol/g vs. 0.037 µmol/g) was also documented. Time effect was observed for phenylethylamine, 1.7-diaminoheptane, tyramine, and methylamine concentrations (*p* < 0.001). Briefly, the level of phenylethylamine decreased after feeding by 30.8% and 52.4% in ZN2 (*p* = 0.038) and ZN4 (*p* < 0.001), respectively. Similarly, the concentration of 1.7-diaminoheptane in the ZS4 group decreased over time (*p* < 0.001; 0.016 µmol/g vs. 0.08 µmol/g). The concentrations of cadaverine and tyramine were 23.1% and 20% lower after feeding in the ZN2 (*p* = 0.045) and control groups (*p* = 0.015), respectively. However, methylamine concentration was on average 2.25 times higher 3 h after feeding in all experimental groups (*p* ≤ 0.001).

The experimental diet did not significantly affect the concentrations of total amines (*p* = 0.099) and putrescine (*p* = 0.176). Importantly, the concentrations of histamine, spermidine, and spermine were below the detection limit.

## 3. Discussion

Both types of zeolites and their contribution to diets did not significantly affect pH in the rumen. The stable pH value after zeolites addition may indicate their buffering properties. Zeolites exert a high affinity for water and active cations, which may influence fermentation and osmotic activity in the rumen, as a consequence [25]. Khachlouf et al. [26] indicated that high content of aluminium and magnesium silicate may influence the buffering capacity of zeolites. The results obtained are in agreement with the study of Bosi et al. [14] on dairy cows supplemented with clinoptilolite (200 g/d). On the contrary, the study of El-Nile et al. [27] documented an increased pH after supplementing Barki goats with natural zeolites (clinoptilolite, 20 g/kg DM diet) and nano-zeolites (0.40 g/kg DM diet). The various actions of zeolites on pH may be dependent on the chemical composition of zeolites, which can act as alkalizers due to the H+ ion exchange capacity [26]. The great importance may also have diet composition, especially with a high contribution of carbohydrates or protein, where zeolites effect could be stronger and more effective. In the present study, the reduction in pH value was noted after feeding animals, which is a normal phenomenon. It is related to the activity of microorganisms and the appearance of the end products of fermentation (organic acids) in the rumen environment [28].

The effect of zeolites on the microorganism population in the gastrointestinal tract of ruminants is still unknown in the literature. In the present study, the protozoa from *Ophryoscolecidae* (*Entodinium* spp., *Diplodinium* spp., *Ophryoscolex* spp.) and *Isotrichidae* (*Isotricha* spp., *Dasytricha* ssp.) families were identified in the rumen of cows. The *Entodinium* genus was a predominant group found in the rumen, while the least numerous ciliates were the *Ophryoscolex* genus. Regardless of sampling time, ZN2 and ZS2 diets reduced the number of total protozoa and *Entodinium* spp., known for decomposing easily digestible carbohydrates (starch). Similarly, the ZS4 diet significantly decreased other populations of ciliates able to utilise soluble carbohydrates (*Dasytricha* ssp.) before feeding. An ambiguous effect of the tested zeolites was observed for fibrolytic protozoa. On the one hand, natural and synthetic zeolites used in different doses decreased the abundance of *Diplodinium* spp. On the other hand, the contribution of 2% of both zeolites in cow diets increased *Ophryscolex* spp., depending on time. On the contrary, in the study of El-Nile et al. [27] on goats, the addition of 20 g natural zeolites (clinoptilolite) and 0.40 g of its nano-form significantly increased the number of *Isotricha* spp. without any effect on other groups. Mahdavirad et al. [15] documented an increased protozoa number in Arabi lambs receiving 2% zeolites in a diet in comparison to the control group. The results obtained from the present study showed that zeolites can act in a type- and dose-dependent manner on different groups of protozoa, utilising both structural and non-structural carbohydrates. Unfortunately, the mechanisms of zeolites’ action on protozoa in the literature are poorly understood. Binding protozoa inside the zeolite structure should be excluded, mainly due to the size of primary cells (20–215 μm depending on the species) in relation to the diameter of the zeolite pores (˂400 pm). However, it is believed that these compounds may affect the ciliate population by impacting the environment of the rumen through their buffering properties. Differences observed in the zeolites’ action on protozoa population may also be derived from the diet composition used in animal studies. In the abovementioned studies, animals received diets with a high contribution of concentrate, including carbohydrates and protein, while in the present study, Jersey cows received diets at a household dose.

In the literature, more studies on zeolites were conducted on different groups of bacteria. Weiβ et al. [29] observed that natural zeolite (clinoptilolite) can be colonised by certain groups of bacteria (*Clostridium*, *Pseudomonas*, *Methanoculleus*) under in vitro conditions, constituting their micro-habitat and affecting their biological activity. Bacterial cell walls are negatively charged physiologically, allowing them to interact with cations on the surface of zeolites [30]. Interestingly, Hrenović et al. [31] demonstrated the positive effect of natural zeolites (clinoptilolite) on the *Acinetobacter junii* (phosphate-accumulating bacteria), while synthetic zeolites of lynde type A had a toxic effect on this culture. Authors indicated that the main mechanism of clinoptilolite action on bacteria was their immobilisation onto the surface, which, in consequence, increased bacterial biomass. In the present study, we did not observe any significant differences regarding the type of zeolites used on the total bacterial population. When interpreting the effects of zeolites’ action on ruminants, the mutual relations between protozoa and bacteria in the rumen should not be omitted. As it is already known, ciliates are predators and engulf bacterial cells, simultaneously regulating the size of their population [32]. However, despite shifts observed in the ruminal protozoa population after zeolite incorporation into the diet, we did not note any significant effect on the total bacteria population in the present study. Goodarzi and Nanekarani [33] documented an increased population of cellulolytic bacteria in a Lori sheep fed a diet with 4% calcic clinoptilolite at 3 and 6 h after feeding without any significant effect on the total bacteria population in the rumen. Authors claimed that the positive effect on cellulolytic bacteria was caused by an increased pH value in the rumen, which improved and favoured the conditions of their growth and development.

Valpotić et al. [25] indicated that zeolites of different origins can influence energy metabolism in ruminants due to changes in the fermentation pattern. The results of the current study showed that the administration of natural and synthetic zeolites did not significantly affect the concentration of SCFAs in the rumen, regardless of the dose used. However, the concentrations of total SCFAs, acetate, propionate, and butyrate were insignificantly higher when animals received ZN4 and ZS4 diets. Similarly, Bosi et al. [14] did not document any significant effect of clinoptilolite on the concentrations of total SCFAs and their types. Roque-Jiménez et al. [34] showed that increasing levels of clinoptilolite (20, 40, 60 g/kg) tended to increase the concentration of SCFAs. McCollum and Galyean [35] noted increased concentration of propionic acid in beef steers fed high-concentrate diets with 2.5% clinoptilolite. Moreover, authors showed that clinoptilolite-rich diets (2.5 and 5% DM) tended to increase the concentration of total SCFAs in the rumen, similar to results obtained from the present study. Grabherr et al. [36] noted an increased proportion of acetate and decreased concentration of propionate and valerate in dairy cows fed a diet with zeolite A in a dose of 10 and 2 g/kg DM per day, without any significant effect on total SCFAs. The differences in the ruminal fermentation pattern can be caused by the chemical structure of zeolites used as well as animal diet composition.

Microorganisms inhabiting the rumen take part in the methanogenesis. Carbon dioxide produced in the carbohydrate fermentation is reduced by methanogens to methane by using hydrogen. Importantly, these microorganisms live in close proximity to protozoa, which, due to the presence of a hydrogenosome (the mitochondrial equivalent) in the cell, are important hydrogen donors in the rumen [37]. Morgavi et al. [38] demonstrated that partial or complete elimination of protozoa inhibited methane production in the rumen. Therefore, any changes in the relationship between protozoa and methanogens may determine the scale of methane production in the rumen. In the study of Wrzosek-Jakubowska and Gworek [23], it was demonstrated that methane can be adsorbed in the channels and chambers of synthetic zeolites (4A and NaY, physical adsorption), using a molecular modelling program. Similar relationships have been documented by Hao et al. [24] for modified clinoptilolite. Other studies have shown that clinoptilolite also has the ability to bind hydrogen (the substrate for the production of methane) in amounts up to 4% of its weight at room temperature [21]. In the present study, the abundance of the methanogen population was unchanged after dietary treatments, with the exception of their lower number in the ZN2 group. Furthermore, despite a lower number of protozoa in zeolite-rich diets, we did not observe any significant effect on methane production, which was contrary to the assumed hypothesis. It is worth noting that gas production is generally related to nutrient degradation and microbial fermentation [39]. An increased cellulolytic activity of the ruminal digesta in cows receiving 2% synthetic zeolites in a diet [16] did not significantly affect ruminal fermentation. Notably, propionic acid is considered a methane antagonist; for that reason, at higher concentrations of this acid, reduced methane production is documented [40]. In the present study, methane production was estimated according to the calculations of SCFAs concentrations [41]. Thus, the lack of relevant changes in the proportions of acetate, propionate, and butyrate after dietary treatments resulted in unaltered methane concentration. Interestingly, an in vitro study of El-Nile et al. [27], showed reduced methane production without any adverse effect of nutrient degradation when increasing level of nano-zeolites was added. The reduced number of protozoa and methanogens population (as a tendency) in the ZN2 group prompts conducting further research on this topic.

Ammonia level is an indicator of the nitrogen degradation in the rumen. The lowest concentration of ammonia was observed for the ZN2 group in both sampling times, which confirms the capacity of zeolites to adsorb harmful substances. Interestingly, zeolites can take up approximately 15% of the ammonia, which prevents excessive absorption of its ions from the rumen [34,42]. Thus, zeolites may improve nitrogen utilisation by gradually releasing excess ammonia and enable bacteria to utilise it for microbial protein synthesis [25]. Zeolites are also considered eco-friendly because of the removal of excess N in faeces and bedding [6,43]. The results of the present study are in agreement with El-Nile et al. [27]. Similarly, in Holstein steers fed a diet containing urea (20 g/kg), a reduced level of ammonia nitrogen in the rumen after 30 g/kg clinoptilolite supplementation was noted [44]. On the other hand, the addition of 1.4% natural zeolites in dairy cows to the TMR diet did not significantly affect the concentration of ammonia N [45].

Biogenic amines are non-volatile nitrogenous compounds of high activity, formed by microbial decarboxylation of amino acids, but also by amination or transamination of ketones and aldehydes [46]. These substances have an impact on protein synthesis, DNA replication, as well as permeability of cell membranes at the cellular level. They are also considered toxic and carcinogenic substances, and their presence in the food is related to health hazards. In the present study non-significant effect of natural and synthetic zeolites of different doses on total amines was noted. Considering individual types of amines, an increased concentration of tryptamine and 1.7-diaminoheptane was observed in the ZS4 group, as well as phenylethylamine in the ZN4 group, depending on time. An increased concentration of some types of amines in the ruminal digesta is unfavourable due to the potential toxicity of biogenic amines on organisms. The in vitro studies of Gokdogan et al. [47] and Özogul et al. [48], examined the effect of natural zeolites (clinoptilolite) on biogenic amine production. It was shown that their effect was dose-dependent and strictly related to the bacterial strains. Interestingly, the addition of 1% zeolite reduced tyramine production by Gram-negative bacteria (especially *E. coli* and *P. aeruginosa*), while 5% zeolite increased their concentration [48]. Moreover, 1% zeolite reduced the accumulation of putrescine and cadaverine by testing Gram-positive bacteria (*S. aureus*, *E. faecalis,* and *L. monocytogenes*), but had a stimulating effect on the tyramine production. Similar dependencies were noted in the present study when ZN4 and ZS4 diets were administered to the animals. Interestingly, the lack of effect on biogenic amine production was observed when cows received ZN2 and ZS2 diets. The results obtained for amines are complementary to the concentration of isoacids in the rumen. It should be underlined that the basal diet for cows contained 10% protein, and perhaps for that reason, the concentrations of biogenic amines were unchanged after 2% zeolite administration. It is thought that the action of zeolites could be more pronounced at higher levels of dietary protein.

## 4. Materials and Methods

Generative artificial intelligence (GenAI) has not been used in this paper.

### 4.1. Animals and Diets

**Figure 1:**
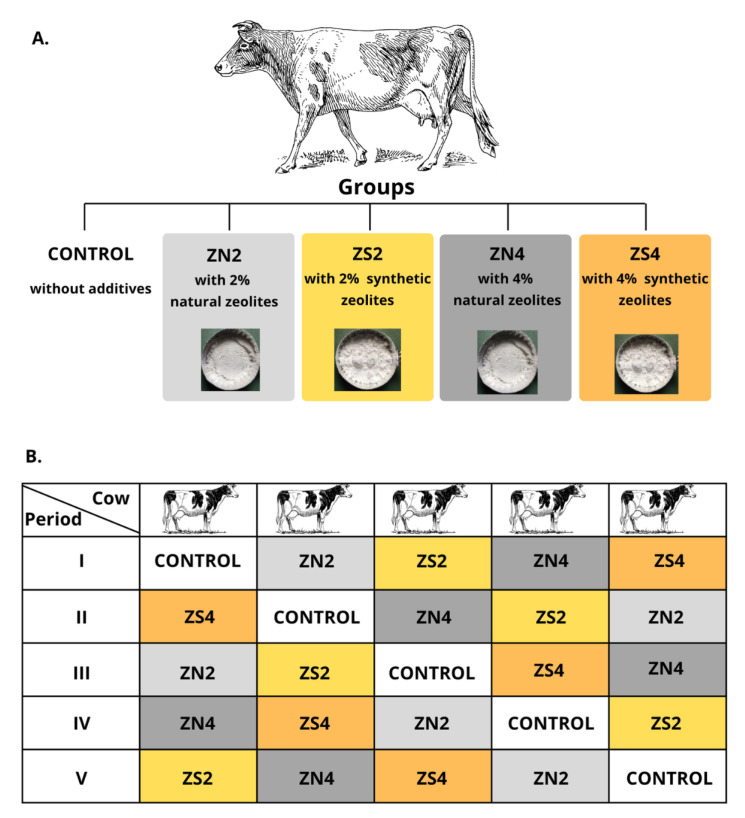
The scheme of the experiment design (prepared using https://canva.com). (**A**). Groups of animals used in the experiment. (**B**). Allocation of cows to specific experimental groups in periods I-V according to 5 × 5 Latin square design.

For this study, five Jersey heifers (two years old, 350 kg live weight) fistulated to the rumen were used in a 5 × 5 Latin square design to compare the effect of various types and doses of zeolites on the ruminal parameters (Figure 1). The experiment consisted of 5 periods with 5 dietary treatments that ensured n = 5 per feeding group. Each experimental period lasted 36 d, including a gradual transition to the diet (14 d), adaptation to the diet (21 d), and sampling (1 d). The animals were housed in the cow room with the individual litter-free stalls equipped with rubber mats. Cows had constant access to the trough, an automatic waterer, and salt licks. The animals were fed twice a day at 7.30 am and 3.30 pm. The basal diet consisted of (kg/d): meadow hay (6), barley meal (0.8), soybean meal (0.2), and mineral-vitamin mixture (Dolfos Dolmix B, 0.04) (Table 1) and was formulated according to the IZ PIB-INRA recommendations to cover the existing needs of the animals [49]. The experimental cows received additionally natural (ZN) or synthetic zeolites (ZS) in a dose of 120 and 240 g/d, which contributed to 2% (ZN2 or ZS2) and 4% (ZN4 or ZS4) of the basal diet, respectively. ZeoFEED (82–86% of clinoptilolite, 200 µm particle size, ZEOCEM, Slovakia) was used as a source of the natural zeolites, while ZP-4A (99% zeolites, 3–5 µm particle size, SILKEM, Slovenia) served as a source of the synthetic zeolites. Feed intake was monitored daily, and all appearing orts were collected during the whole experiment.

Feed samples (barley meal, soybean meal, both types of zeolites, meadow hay) were collected throughout the whole experiment. The chemical composition of each ingredient was analysed according to the AOAC methods [50]. The following components were determined: dry matter (DM, 934.01), crude protein (CP, 954.01), crude fat (CF, 930.09), crude ash (CA, 930.05), crude fibre (978.10), neutral detergent fibre (NDF, 2002.04), acid detergent fibre (ADF, 973.18) and acid detergent lignin (ADL, 973.18). The concentration of non-fibrous carbohydrate (NFC) in cow diets was determined according to the following formula [51]:

$$NFC=100-\left(NDF+CP+CF+CA\right).$$

### 4.2. Characterisation of Zeolites

Both types of zeolites belong to the technological additives, mainly used as binders, anti-caking agents, and coagulants (according to the manufacturer’s information).

Clinoptilolite is a hydrated aluminosilicate, (Na_4_K_4_)(Al_4_Si_4_O_96_)·24H_2_O. The mineral composition of this zeolite and its purity depend largely on the place where it was deposited. Furthermore, clinoptilolite has been classified by the European Union as a feed additive for animals. In the United States, this mineral has been granted ‘safe’ status [9].

Zeolite 4A is a synthetic sodium aluminosilicate (Na_2_O∙Al_2_O_3_∙2SiO_2_∙4.5H_2_O), also known as E554. It is obtained by the reaction of aluminium sulphate and sodium silicate, followed by precipitation, or by the reaction of sodium meta-silicate, metabisulphite, and aluminium sulphate by steam heating. This zeolite is listed in a Commission Regulation (EU) No 231/2012 of 9 March 2012 [52] as an authorised food additive and classified as an additive other than colours and sweeteners. More detailed characteristics of zeolites used in the present study are presented in Table 6.

### 4.3. Sampling Procedure

The ruminal fluid (on average about 400 mL) was collected from different regions of the rumen (middle and ventral sacs) by using a copper tube with numerous holes connected to a syringe, precisely mixed, and filtered through a fourfold sterile surgical gauze. The ruminal fluid samples were used to determine pH value, protozoa number, SCFAs, and ammonia concentrations.

Solid and liquid fractions of ruminal digesta (on average about 400 g) were collected by hand from dorsal and ventral sacs of the rumen to obtain representative samples, precisely mixed, collected into sterile plastic tubes, and frozen at −24 °C (amines analysis) and −80 °C (microbiological analysis).

The samples of ruminal fluid and digesta were taken before morning feeding (0 h) and 3 h after feeding to observe changes over time.

### 4.4. Determination of Protozoa Number

A 5 mL sample of each ruminal fluid was preserved with a 10 mL sample of 4% aqueous formaldehyde solution and stored at 4 °C in sealed plastic containers until analysis. Each sample was counted under a light microscope in 3 replications. Morphological criteria (number and size of ciliary zones, number and location of contractile vacuoles, number of spines, size and shape of cell) described by Dehority [53] and followed by Miltko et al. [54] served as a basis to identify protozoa numbers from *Ophryoscolecidae* and *Isotrichidae* families.

### 4.5. Determination of Bacteria and Methanogens Abundance

The isolation of genomic DNA from the tested samples (up to 250 mg) was carried out using the QIAamp PowerFecal Pro DNA kit on the basis of the protocol enclosed by the manufacturer (Qiagen, Hilden, Germany). The quality of the isolated DNA was tested by electrophoresis on 1% agarose gel stained with ethidium bromide, and the purity of the DNA was determined by the NanoDrop 2000 spectrophotometer (Thermo Scientific, Wilmington, DE, USA). The samples with the isolated DNA were stored at −80 °C. The qPCR analyses were performed on the Rotor-Gene Q thermocycler (Qiagen, Germany). During analyses, different pairs of bacterial and methanogen starters were tested after prior testing on the BLAST platform (https://www.ncbi.nlm.nih.gov/tools/primer-blast/, accessed on 9–11 February 2024), and the most specific ones were selected (Genomed, Table 7).

The qPCR reaction was performed in a 20 μL volume using: 13.5 μL of sterile water, 4 μL of the master mix 5× HOT FIREPol^®^ EvaGreen^®^ Mix Plus (no rox) (Solis Biodyne, Tartu, Estonia), 1.5 μL of the tested DNA, and 0.5 μL of each starter (10 μM/μL). The regular qPCR conditions for total bacterial counts were: 95 °C for 10 s (denaturation), 55 °C for 20 s (annealing) and 72 °C for 10 s (extension) (30 cycles), with the exception for the 12 min at 95 °C of the initial activation of polymerase in the first cycle and the 7 min at 72 °C of the extension in the last cycle.

The other regular qPCR conditions were established for the total methanogens: 95 °C for 10 s (denaturation) and 60 °C for 30 s (annealing and extension) (35 cycles), except for the 12 min at 95 °C of the initial activation of polymerase in the first cycle and the 5 min at 72 °C of the extension in the last cycle.

The analysis of qPCR for bacteria and methanogens was performed in 3 replications.

### 4.6. Determination of SCFAs Concentrations and Methane Production

**Figure 2:**
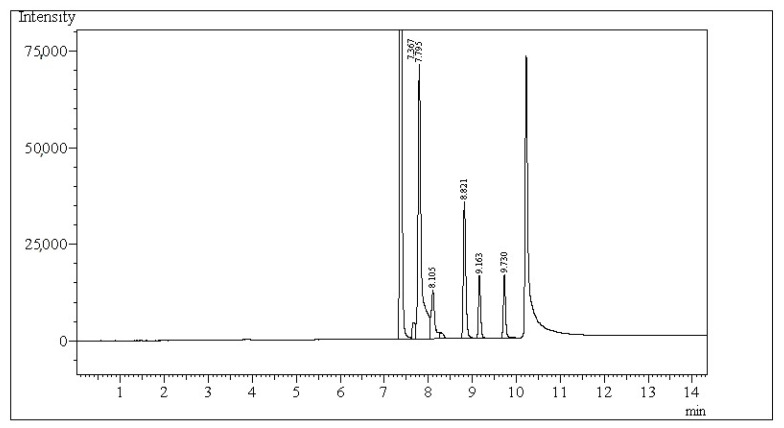
The chromatogram presents SCFAs in the following order: acetic acid (RT, retention time—7.367 min), propionic acid (RT—7.795 min), iso-butyric acid (RT—8.105 min), butyric acid (RT—8.821 min), iso-valeric acid (RT—9.163 min), and valeric acid (RT—9.730 min).

A 5 mL of each ruminal fluid sample for SCFAs analysis was treated with 0.5 mL of 85% formic acid, centrifuged (11,000× *g* for 25 min, 4 °C), and stored in the refrigerator until analysis. The concentration of SCFAs was determined by gas chromatography. The Shimadzu GC-2010 chromatograph (Tokyo, Japan) equipped with a ZB-WAX capillary column (30 m length, 0.25 mm i.d. × 0.25 µm, Zebron, Phenomenex, Torrance, CA, USA) and flame ionisation detector (FID) was used to perform SCFAs analysis according to the method described by Miltko et al. [57]. The temperature of the injector and FID was 250 and 280 °C, respectively. Helium was a carrier gas. A 4-methylvaleric acid served as an internal standard (Sigma-Aldrich Co., St. Louis, MO, USA). The concentration of SCFAs in a 1 µL of experimental sample was measured according to the set column temperature programme at a split ratio of 10:1. The initial temperature of the column was 80 °C, maintained for 1 min. Then, the temperature increased every 15 °C per min, finally to 220 °C maintained for 4 min. Chromatograms obtained have been analysed for specific peaks of fatty acids (FAs, Figure 2). The identification of selected FAs was possible due to the provided standards (acetic acid, propionic acid, butyric acid, iso-butyric acid, valeric acid, and iso-valeric acid). All standards were purchased from Sigma-Aldrich Co. (St. Louis, MO, USA). Each peak of FAs was integrated by using GC software 112 (LabSolutions, Shimadzu, Tokyo, Japan) to obtain the field of peak area.

Methane concentration in the rumen was calculated on the basis of acetic (C2), propionic (C3), and butyric acids (C4) according to the following formula [41]:

$$Methane=\frac{\left(1.8\times C2-1.1\times C3+1.6\times C4\right)}{4}$$

### 4.7. Analysis of Ammonia Concentration

The ammonia concentration in the ruminal fluid was spectrophotometrically determined based on the reaction of ammonium ions with Nessler’s reagent according to the method described by Taciak et al. [58]. The absorbance was measured at 425 nm using a SpectraMax iD3 microplate reader (Molecular Devices, San Jose, CA, USA). The ammonium chloride solution was used to prepare a standard curve, and on this basis, the concentration of ammonia was calculated.

### 4.8. Analysis of Amines Concentration

The amines concentration in the ruminal digesta was determined according to the HPLC method [59] after derivatization with 1% dansyl chloride in acetone, using SEP-PAK C18 solid-phase extraction cartridges (6 mL, 500 mg Waters Ltd., Watford, Hertfordshire, UK). The separation was performed using a Finnigan Surveyor Plus liquid chromatograph (Thermo Scientific, San Jose, CA, USA) equipped with a photodiode array detector set to 240 nm. Waters Symmetry Shield RP_18_ guard column (20 × 3.9 mm id., particle size 5 µm) and Waters Symmetry Shield RP_18_ column (150 × 3.9 mm id., particle size 5 µm) were used for chromatographic separation. Heptylamine served as an internal standard. Amines were identified and quantified according to the previously prepared standard curves of pure compounds.

### 4.9. Statistical Analyses

The results obtained from the present study were presented as means with the standard error of the mean (SEM). The Shapiro–Wilk test was used to check the normality of the data. The results with abnormal distribution were transformed into logarithms. Levene’s test was used to assess the homogeneity of variances. The results obtained were subjected to repeated measures ANOVA followed by Tukey’s HSD post hoc test. The main effects were as follows: diet (CON, ZN2, ZS2, ZN4, ZS4), sampling time (0 h, 3 h), and their interactions. Additionally, the effect of animal (1, 2, 3, 4, 5) and period (I, II, III, IV, V) was also verified. The significances between means were determined at *p* < 0.05, and all appearing trends were discussed at 0.05 < *p* < 0.10 (TIBCO^®^ Software Inc., Statistica^TM^, version 13.3, San Ramon, CA, USA). The results in the tables are presented as raw data before logarithmic transformation.

## 5. Conclusions

The results obtained from the present study showed that zeolites can act both in a type- and dose-dependent manner. Both ZN2 and ZS2 diets significantly reduced the number of total protozoa and *Entodinium* spp., *Diplodinium* spp., while the increased population of *Ophryoscolex* spp., depending on sampling time. Furthermore, the usage of different types and doses of zeolites in cow diets did not significantly affect pH, bacteria, and methanogens population, as well as SCFAs and methane production in the rumen. Importantly, differences in the response of studied parameters to both types of zeolites’ action can be a result of their chemical composition and, consequently, their physicochemical properties.

The addition of 4% zeolites in cow diets reduced feed intake and increased concentration of ammonia and specific amines (tryptamine, 1.7-diaminoheptane, phenylethylamine), which can be not profitable for breeders and unfavourable for animal health.

Further research on a larger group of animals is necessary to propose the mechanisms of zeolites’ action on the microorganisms population and nutrient digestion in the rumen in the context of limiting the methane production. The usage of advanced computational chemistry tools, including Material Studio and SciGress computer programs, is extremely important to model the sorption and desorption of chemical compounds on the zeolites in the rumen, as well as to visualise and model zeolites to determine their unique properties in the future.

## Figures and Tables

**Table 1:** Composition of cow diets (adopted from Majewska et al. [16]).

Item	Control	Natural Zeolites (ZN)		Synthetic Zeolites (ZS)	
		ZN2	ZN4	ZS2	ZS4
Components (g/kg DM)					
Meadow hay	855	840	826	841	827
Barley meal	111	109	107	109	107
Soybean meal	28.2	27.7	27.2	27.7	27.3
Dolfos ^1^	6.18	6.07	5.96	6.07	5.97
ZeoFEED	-	17.6	34.6	-	-
ZP-4A	-	-	-	16.7	32.8
Chemical composition (g/kg DM)					
DM	897	897	898	896	896
Crude protein ^2^	94.3	92.7	91.2	92.7	91.2
Crude fat	23.0	22.6	22.2	22.6	22.2
Crude ash	37.6	37.0	36.3	37.0	36.3
Crude fibre	271	266	262	266	262
NDF	599	588	579	588	579
ADF	352	346	340	346	340
ADL	59.9	58.9	57.9	58.9	57.9
NFC	241	237	233	237	233
Nutrient intake (g/d)					
DM	6312	6361	6385	6487	6184
Crude protein	664	657	661	659	629
Crude fat	162	160	161	161	154
Crude ash	265	262	264	263	251
Crude fibre	1905	1886	1895	1890	1805
NDF	4214	4172	4193	4181	3994
ADF	2478	2453	2465	2459	2348
ADL	422	418	420	419	400
NFC	1697	1680	1684	1684	1608

DM—dry matter, NDF—neutral detergent fibre, ADF—acid detergent fibre, ADL—acid detergent lignin, NFC—non-fibrous carbohydrate. ^1^ Dolfos DOLMIX B consisted of: calcium carbonate; sodium chloride; calcium-magnesium carbonate; mono-calcium phosphate; magnesium oxide; glycerol; UI: vit. A 700,000; vit. D3 140,000; mg: vit. E 1650; niacin 500; DL-α-tocopherol 1500; trace elements mg: copper 30; manganese 60; zinc 1000; selenium 30; g: sulfur 5.5; calcium 253; sodium 80; magnesium 30; phosphorus 10. ^2^ expressed as N × 6.25.

**Table 2:** Protozoa number in the ruminal fluid of cow (×10^4^/mL).

Protozoa Number	Diet (D)	Sampling Time (T)		SEM	*p*-Value		
		0 h	3 h		D	T	D × T
Total protozoa	Control	18.16 ^A^	18.72 ^A^	0.586	0.023	0.626	0.054
	ZN2	15.62 ^AB^	11.01 ^B^				
	ZS2	11.50 ^B^	13.75 ^AB^				
	ZN4	14.01 ^AB^	13.82 ^AB^				
	ZS4	17.32 ^AB^	17.60 ^AB^				
*Entodinium*	Control	12.75 ^AB^	14.07 ^A^	0.576	0.021	0.975	0.015
	ZN2	11.08 ^AB^	6.88 ^B^				
	ZS2	6.98 ^B^	10.25 ^AB^				
	ZN4	9.06 ^AB^	10.37 ^AB^				
	ZS4	14.83 ^A^	13.23 ^AB^				
*Diplodinium*	Control	1.98 ^A^	2.07 ^A^	0.086	<0.001	0.015	<0.001
	ZN2	1.19 ^B^	1.25 ^A^				
	ZS2	1.04 ^B^	0.66 ^B^				
	ZN4	1.16 ^B^_X_	0.61 ^B^_Y_				
	ZS4	0.38 ^C^	0.56 ^B^				
*Ophryoscolex*	Control	0.15 ^B^_X_	0.02 ^C^_Y_	0.012	<0.001	<0.001	<0.001
	ZN2	0.13 ^B^	0.15 ^A^				
	ZS2	0.34 ^A^_X_	0.14 ^AB^_Y_				
	ZN4	0.18 ^B^	0.13 ^AB^				
	ZS4	0.11 ^B^	0.08 ^B^				
*Isotricha*	Control	0.63	0.58	0.026	0.206	0.969	0.025
	ZN2	0.57	0.52				
	ZS2	0.74	0.51				
	ZN4	0.70	0.71				
	ZS4	0.46 _Y_	0.77 _X_				
*Dasytricha*	Control	2.64 ^AB^	1.98	0.100	0.961	0.279	<0.001
	ZN2	2.64 ^AB^	2.20				
	ZS2	2.40 ^AB^	2.19				
	ZN4	2.90 ^A^	2.01				
	ZS4	1.54 ^B^_Y_	2.96 _X_				

D—effect of diet, T—effect of sampling time, D × T—diet and sampling time interaction effect, SEM—standard error of mean. ^A,B,C^—means with different letters in a column differ significantly at *p* ≤ 0.05 between diet (control, ZN2, ZS2, ZN4, ZS4). _X,Y_—mean with different letters in a row differ significantly at *p* ≤ 0.05 between sampling times (0, 3 h).

**Table 3:** The abundance of microorganisms in the ruminal digesta of cow (log_10_ rrs copies/g).

Item	Diet (D)	Sampling Time (T)		SEM	*p*-Value		
		0 h	3 h		D	T	D × T
Total bacteria	Control	12.47	12.32	0.279	0.992	0.077	0.560
	ZN2	12.43	12.33				
	ZS2	12.40	12.38				
	ZN4	12.45	11.58				
	ZS4	12.39	11.47				
Total methanogens	Control	8.08	7.98	0.017	0.930	0.009	0.806
	ZN2	8.09	7.97				
	ZS2	8.03	7.98				
	ZN4	8.06	8.03				
	ZS4	8.03	7.94				

D—effect of diet, T—effect of sampling time, D × T—diet and sampling time interaction effect, SEM—standard error of mean.

**Table 4:** pH, SCFAs (mM/100 mL), methane (mM/100 mL) and ammonia (mM/L) concentrations in the rumen.

Item	Diet (D)	Sampling Time (T)		SEM	*p*-Value		
		0 h	3 h		D	T	D × T
pH	Control	7.24	6.92	0.031	0.176	<0.001	0.958
	ZN2	7.27 _X_	6.94 _Y_				
	ZS2	7.17 _X_	6.93 _Y_				
	ZN4	7.12 _X_	6.83 _Y_				
	ZS4	7.07 _X_	6.83 _Y_				
Total SCFA	Control	7.19	7.19	0.120	0.490	0.989	0.791
	ZN2	7.13	7.18				
	ZS2	7.37	7.23				
	ZN4	7.45	7.83				
	ZS4	8.04	7.75				
Acetic acid	Control	4.99	4.90	0.079	0.516	0.329	0.744
	ZN2	4.93	4.90				
	ZS2	5.10	4.88				
	ZN4	5.14	5.30				
	ZS4	5.56	5.22				
Propionic acid	Control	1.23	1.21	0.027	0.314	0.698	0.955
	ZN2	1.21	1.20				
	ZS2	1.25	1.22				
	ZN4	1.30	1.35				
	ZS4	1.42	1.38				
Butyric acid	Control	0.63 _Y_	0.75 _X_	0.020	0.825	<0.001	0.596
	ZN2	0.63	0.77				
	ZS2	0.69	0.79				
	ZN4	0.66	0.86				
	ZS4	0.70	0.81				
Valeric acid	Control	0.09	0.11	0.003	0.809	0.017	0.598
	ZN2	0.09	0.11				
	ZS2	0.10	0.12				
	ZN4	0.10	0.10				
	ZS4	0.10	0.10				
Isoacids ^1^	Control	0.25	0.21	0.005	0.786	<0.001	0.840
	ZN2	0.27 _X_	0.21 _Y_				
	ZS2	0.25 _X_	0.21 _Y_				
	ZN4	0.26 _X_	0.22 _Y_				
	ZS4	0.27 _X_	0.23 _Y_				
Methane	Control	2.16	2.17	0.034	0.607	0.830	0.657
	ZN2	2.14	2.18				
	ZS2	2.22	2.18				
	ZN4	2.22	2.36				
	ZS4	2.39	2.30				
Ammonia	Control	3.52 ^AB^	2.27 ^B^	0.144	0.007	<0.001	0.138
	ZN2	2.35 ^B^	2.19 ^B^				
	ZS2	3.16 ^AB^	2.53 ^BC^				
	ZN4	3.14 ^AB^	2.86 ^B^				
	ZS4	4.43 ^A^	3.97 ^A^				

D—effect of diet, T—effect of sampling time, D × T—diet and sampling time interaction effect, SEM—standard error of mean. ^1^ sum of iso-butyric and iso-valeric acids. ^A,B,C^—means with different letters in a column differ significantly at *p* ≤ 0.05 between diet (control, ZN2, ZS2, ZN4, ZS4). _X,Y_—mean with different letters in a row differ significantly at *p* ≤ 0.05 between sampling times (0, 3 h).

**Table 5:** The concentrations of biogenic amines in the ruminal digesta of cow (µmol/g).

Item	Diet (D)	Sampling Time (T)		SEM	*p*-Value		
		0 h	3 h		D	T	D × T
Total amines	Control	0.091	0.099	0.0028	0.099	0.378	0.492
	ZN2	0.109	0.099				
	ZS2	0.104	0.111				
	ZN4	0.116	0.123				
	ZS4	0.119	0.122				
Methylamine	Control	0.007 _Y_	0.016 _X_	0.0009	0.494	<0.001	0.100
	ZN2	0.006 _Y_	0.015 _X_				
	ZS2	0.005 _Y_	0.019 _X_				
	ZN4	0.004 _Y_	0.017 _X_				
	ZS4	0.005 _Y_	0.017 _X_				
Tryptamine	Control	0.007	0.007 ^B^	0.0002	0.014	0.500	0.460
	ZN2	0.007	0.007 ^B^				
	ZS2	0.008	0.008 ^AB^				
	ZN4	0.007	0.009 ^AB^				
	ZS4	0.009	0.009 ^A^				
Phenylethylamine	Control	0.011 ^B^	0.010	0.0006	0.007	<0.001	0.009
	ZN2	0.013 ^AB^_X_	0.009 _Y_				
	ZS2	0.010 ^B^	0.008				
	ZN4	0.021 ^A^_X_	0.010 _Y_				
	ZS4	0.009 ^B^	0.008				
Putrescine	Control	0.019	0.019	0.0005	0.176	0.239	0.307
	ZN2	0.020	0.018				
	ZS2	0.016	0.016				
	ZN4	0.022	0.019				
	ZS4	0.016	0.016				
Cadaverine	Control	0.036	0.037	0.0021	0.040	0.578	0.387
	ZN2	0.052 _X_	0.040 _Y_				
	ZS2	0.055	0.050				
	ZN4	0.051	0.058				
	ZS4	0.060	0.060				
1.7-diaminoheptane	Control	0.006 ^B^	0.006	0.0004	<0.001	<0.001	<0.001
	ZN2	0.006 ^B^	0.006				
	ZS2	0.006 ^B^	0.006				
	ZN4	0.006 ^B^	0.006				
	ZS4	0.016 ^A^_X_	0.008 _Y_				
Tyramine	Control	0.005 _X_	0.004 _Y_	0.0001	0.964	<0.001	0.387
	ZN2	0.005	0.004				
	ZS2	0.004	0.004				
	ZN4	0.005	0.004				
	ZS4	0.004	0.004				

D—effect of diet, T—effect of sampling time, D × T—diet and sampling time interaction effect, SEM—standard error of mean. ^A,B^—means with different letters in a column differ significantly at *p* ≤ 0.05 between diet (control, ZN2, ZS2, ZN4, ZS4). _X,Y_—mean with different letters in a row differ significantly at *p* ≤ 0.05 between sampling times (0, 3 h).

**Table 6:** Characteristics of zeolites used in cow diets (according to the product information).

Parameters	Natural Zeolites ^1^	Synthetic Zeolites ^2^
Ingredients	≥90% clinoptilolite	>99% zeolite 4A
Chemical structure ^3^	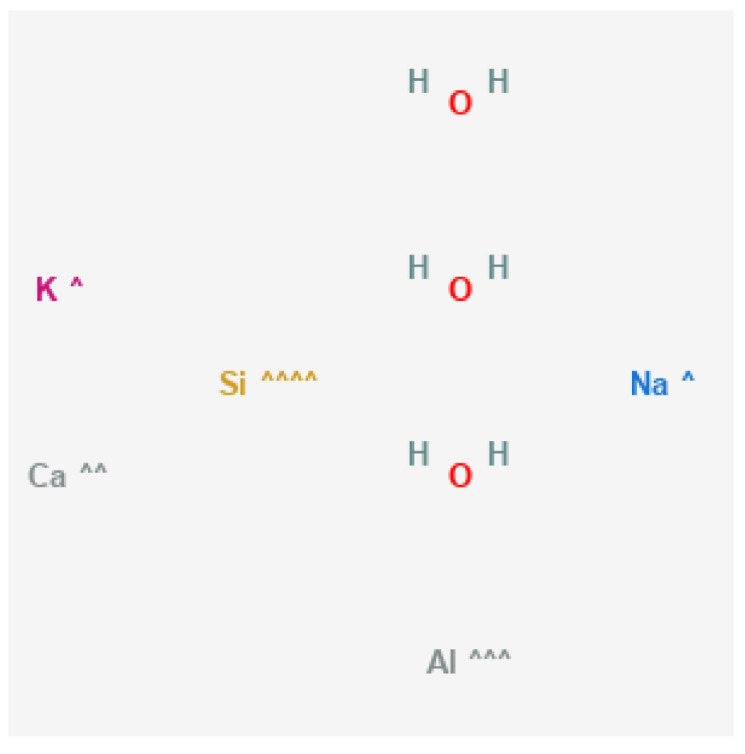	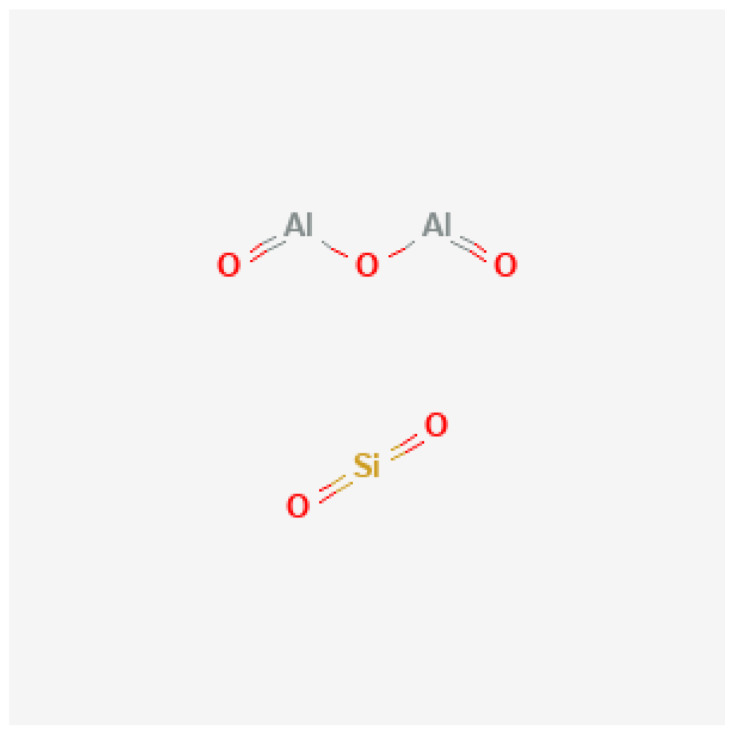
Framework type ^4^	HEU 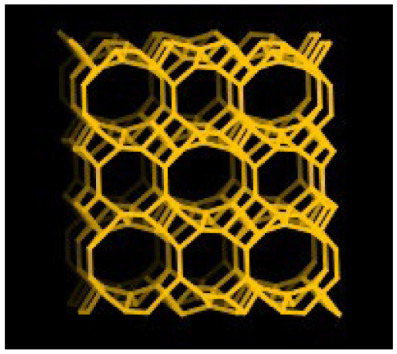	LTA 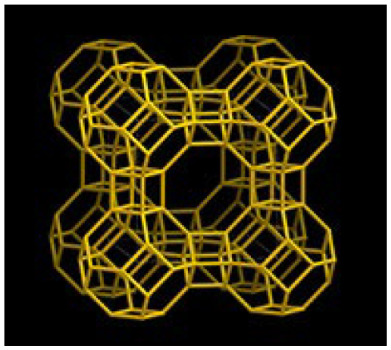
Chemical composition (%)		
SiO_2_	63.2	29.0
Al_2_O_3_	12.3	32.5
Fe_2_O_3_	1.45	-
CaO	2.85	-
Na_2_O	-	18.0
Heavy metals (ppm)		
Pb	12.3	<0.33
Cd	0.021	<0.014
Hg	0.007	<0.010
As	1.24	<0.20

^1^ ZeoFeed^®^ from ZEOCEM, Bystré, Slovakia (CAS: 12173-10-3); ^2^ ZP-4A from SILKEM, Slovenia (CAS: 1318-02-1; EC: 930-915-9); ^3^ chemical structure of zeolites was adopted from https://pubchem.ncbi.nlm.nih.gov (accessed on 26 August 2025); ^ the valency of elements, e.g., K ^ (potassium with valence I), Na ^ (sodium with valence I), Ca ^^ (calcium with valence II), Al ^^^ (aluminium with valence III) and Si ^^^^ (silicon with valence IV); ^4^ framework type of zeolites was adopted from https://www.iza-structure.org/databases (accessed on 26 August 2025).

**Table 7:** Primers for qPCR assay.

Target Species	Primer Sequence (5′→3′)	Amplicon	References
Total bacteria	F: GTGSTGCAYGGYTGTCGTTCA	150 bp	[55]
	R: ACGTCRTCCMCACCTTCCTC		
Total methanogens	F: CCGGAGATGGAACCTGAGAC	164 bp	[56]
	R: CGGTCTTGCCCAGCTCTTATTC		

## Data Availability

The data presented in this study are available on request from the corresponding author.

## Abbreviations

The following abbreviations are used in this manuscript:

ADF	Acid detergent fibre
ADL	Acid detergent lignin
CA	Crude ash
CF	Crude fat
CP	Crude protein
DM	Dry matter
FAs	Fatty acids
FID	Flame ionisation detector
IPCC	Intergovernmental Panel on Climate Change
NDF	Neutral detergent fibre
NFC	Non-fibrous carbohydrate
RT	Retention time
SCFAs	Short-chain fatty acids
SEM	Standard error of mean
ZN	Natural zeolites
ZS	Synthetic zeolites
